# Identifying subgroups of individuals undergoing metabolic bariatric surgery based on behavioral and psychosocial factors: A latent profile analysis

**DOI:** 10.1371/journal.pone.0352252

**Published:** 2026-06-24

**Authors:** Ellen A. M. Kuipers, Josien G. Timmerman, Marc J. van Det, Marloes Vermeer, Miriam M. R. Vollenbroek - Hutten

**Affiliations:** 1 Department of Surgery, Hospital Group Twente, Almelo/Hengelo, The Netherlands; 2 Biomedical Signals and Systems, Faculty of Electrical Engineering, Mathematics and Computer Science, University of Twente, Enschede, The Netherlands; 3 ZGT Academy, Hospital Group Twente, Almelo/Hengelo, The Netherlands; 4 Board of Directors, Medisch Spectrum Twente, Enschede, The Netherlands; Athens Medical Group, Psychiko Clinic, GREECE

## Abstract

**Purpose:**

We performed a latent profile analysis (LPA) to identify subgroups in the population undergoing metabolic bariatric surgery (MBS), based on various behavioral and psychosocial characteristics. Gaining deeper insights into the variability among preoperative MBS patients may be helpful in facilitating tailored care that addresses the specific needs of each profile, in relation to physical, emotional, and psychological functioning.

**Methods:**

Within a prospective cohort study, demographics and questionnaires regarding general health, quality of life, social support, problematic eating behavior (i.e., emotional eating, external eating, restrained eating, disordered eating) and depressive symptoms were collected preoperatively. LPA was performed to identify homogeneous subgroups.

**Results:**

Out of 272 patients, four distinctive profiles were derived. The profiles were: (1) ‘*higher psychosocial and physical functioning profile’* (n = 122, 45%): no deficits on all domains; (2) *‘higher psychosocial and lower physical functioning’* (n = 53, 20%): moderate impairments in physical functioning and problematic eating behavior, with no to mild impairments in emotional wellbeing and high scores on social support; (3) ‘*lower psychosocial functioning and stable physical health’* (n = 36, 13%): mild impairments in physical functioning, with moderate levels of depressive symptoms and problematic eating behavior; (4) ‘*lower psychosocial and physical functioning profile’* (n = 61, 22%): moderate deficits in general health, problematic eating behavior, depressive symptoms, social support, and quality of life.

**Conclusion:**

These findings suggest that subgroups can be identified, differing in behavioral and psychosocial characteristics, including psychological well-being, eating behavior, social support, and physical and emotional functioning among patients prior to MBS. Future longitudinal studies are necessary to establish the prognostic validity of these subgroups.

## Introduction

In the Netherlands, nearly 12,000 patients with severe obesity undergo metabolic bariatric surgery (MBS) annually [1]. Patients undergo MBS to achieve sustainable weight loss, which often leads to the reduction or resolution of obesity-related comorbidities, and to improvements in quality of life [2]. Optimal results after MBS are dependent on patient commitment to lifestyle changes, such as adopting healthier eating and physical activity habits [3]. Although the goal of MBS is similar for most individuals, namely health improvements such as the reduction of comorbidities, enhanced mobility, and increased longevity [4–6], baseline behavioral and psychosocial characteristics differ between individuals prior to the start of their MBS trajectory. This heterogeneity (i.e., the natural variability between patients) suggests the potential to identify more homogeneous subgroups within the MBS population, which may help in tailoring treatment strategies accordingly. For example, individuals exhibiting problematic eating behavior might receive additional support from a dietitian or psychologist.

Despite variability in patients’ lifestyle behaviors, this heterogeneity has received limited attention in the care provided to patients prior to MBS. To the best of our knowledge, only three studies have analyzed patient subtypes prior to MBS [7–9]. Claes et al. and Müller et al. subtyped patients based on temperament features (such as neuroticism, extraversion, and agreeableness), which refer to biologically based individual differences in behavioral tendencies [7,8]. Extending this approach, Schäfer et al. incorporated emotion dysregulation and eating behavior into existing temperament traits subtyping models, revealing subgroups prior to MBS with distinct psychological deficit patterns [9]. While these studies provide insight into psychological subtypes within the MBS population, their focus is predominantly unidimensional and centers on factors that are difficult to change. This highlights the need for a more multidimensional approach that not only captures a broader range of behavioral and psychosocial factors but also emphasizes domains that are potentially amenable to change. Incorporating these domains would broaden the characterization of preoperative behavioral and psychosocial variation that may provide direction for the focus of preoperative support programs.

Previous research has examined several modifiable behavioral and psychosocial factors in relation to weight loss outcomes after MBS, including physical activity [10,11], problematic eating behavior [11–15], and ongoing psychopathology [16,17]. However, when examined in isolation, psychosocial predictors of postoperative outcomes often show inconsistent results or only modest associations with outcomes [16]. At the same time, previous literature suggests that psychosocial factors, such as social support, may influence depression, eating behavior, and weight loss outcomes [18]. These findings indicate that behavioral and psychosocial characteristics are unlikely to operate independently. This aligns with the biopsychosocial model, which conceptualizes health outcomes as the result of dynamic interactions between psychological, behavioral, and biological domains. Within this framework, interrelated psychological and behavioral characteristics may cluster within individuals, forming distinct patterns of vulnerability and resilience. A multidimensional approach incorporating multiple behavioral and psychosocial domains may therefore provide a more comprehensive understanding of heterogeneity within the MBS population. Identifying subgroups of patients with comparable behavioral and psychosocial characteristics may, in turn, contribute to a more clinically relevant understanding of preoperative functioning.

Latent profile analysis (LPA) might be a useful technique in this respect, as it enables the identification of unobserved (latent) subgroups of individuals based on similar response patterns across continuous variables. LPA has gained considerable popularity in recent years [19], especially in studies focusing on mental health and eating disorders. For example, LPA has been used to identify distinct subgroups in individuals with eating disorders and comorbid psychopathology [20], those with compulsive eating and obesity [21], and individuals experiencing depression and anxiety [22]. Within our study population of preoperative MBS patients, LPA may provide novel insights into subgroups with specific behavioral and psychosocial patterns, which may be helpful in developing intervention strategies that align with the characteristics of these subgroups. This approach may also enhance our understanding of why outcomes after MBS vary between individuals.

Therefore, this study aims to identify subgroups within the preoperative MBS population based on different behavioral and psychosocial characteristics. In addition, potential differences in patient characteristics such as age, gender, BMI, obesity-related comorbidities, education level, employment status, marital status, smoking habits, and alcohol consumption between the subgroups were explored.

## Methods

### Study design

This study used baseline data from a prospective observational cohort registry about predictors for outcomes after MBS. The study is conducted at the obesity center of Hospital Group Twente (ZGT), Almelo/Hengelo, and was approved by the local medical ethics committee (local registration number: K19-23, Netherlands trial registration number: NL-9228). All study participants provided written informed consent.

### Participants and data collection

We identified patients scheduled for MBS and recruited them at the outpatient clinic between July 8, 2019 and December 10, 2022. Data used for analysis in this study were collected from patients who were qualified for primary MBS. The criteria of the ‘International Federation for the Surgery of Obesity and Metabolic Disorders’ were used to qualify for MBS [23]. Patients who underwent revisional MBS and patients with an insufficient understanding of the Dutch spoken and written language were excluded.

Demographics and baseline data were obtained from medical records and questionnaires. Data extracted from medical records included age, gender, weight and height (measured during physical examination at the outpatient clinic before MBS), body mass index (BMI), and the presence (yes/no) of obesity-related comorbidities (type 2 diabetes mellitus (T2DM), hypertension, gastroesophageal reflux disease (GERD), osteoarthrosis, obstructive sleep apnea syndrome (OSAS) and dyslipidemia). Demographics collected via questionnaire included highest level of education, employment status, marital status, alcohol consumption (units/week), and smoking habits (yes/no). Employment status was categorized into ‘student’, ‘working’, ‘unemployed’, or ‘other’. Marital status was categorized as ‘single’, ‘in a relationship (not cohabiting)’, ‘cohabiting’, ‘married’, ‘divorced’, or ‘widowed’. Behavioral and psychosocial questionnaires included problematic eating behavior (i.e., emotional eating, restrained eating, external eating, disordered eating), depression, social support, quality of life, and general health. All questionnaires were self-administered and collected electronically using Castor EDC.

### Lifestyle behavior measures

*Eating behavior –* For the assessment of eating behavior, the Dutch Eating Behavior Questionnaire (DEBQ) and the Eating Disorder Examination Questionnaire (EDE-Q) were used [24,25]. The DEBQ consists of 33 questions assessing three dimensions of eating behavior: restrained eating, emotional eating, and external eating. Restrained eating refers to conscious efforts to restrict food intake. Emotional eating pertains to the tendency to eat in response to negative emotions, such as stress or sadness. External eating focuses on eating in response to external cues. DEBQ items are rated on a scale from 1 (never) to 5 (very often), where higher scores signify stronger endorsement of the eating behavior. Mean scores for each subscale were calculated (range: 1–5). Eating disorder symptoms were assessed with the EDE-Q. It comprises a series of questions covering different dimensions of disordered eating behavior, namely eating concern, shape concern, weight concern, and restrained eating. Respondents reflected on their experiences over the preceding 28 days, rating the frequency or intensity of each item on a seven-point Likert scale ranging from 0 (absent) to 6 (present every day or to an extreme degree). The mean score was calculated, with a higher score indicating more severe eating psychopathology (range: 0–6).

*Depression –* Patients completed the Beck Depression Inventory (BDI) to evaluate depressive symptoms [26]. The questionnaire consists of 21 items, each scored from 0 to 3, yielding a total score ranging from 0 to 63. Higher scores indicate more severe depressive symptomatology. Conventionally, a score below 10 indicates the absence or minimal presence of depressive symptoms; scores from 10 to 14 indicate borderline depression; scores from 15 to 20 reflect mild symptoms, scores from 21 to 30 suggest moderate symptoms; scores from 31 to 40 indicate severe symptoms, and scores above 40 very severe depressive symptoms.

*Social support –* The Multidimensional Scale of Perceived Social Support (MSPSS) was used to assess an individual’s perception of social support from three sources: family, friends, and significant others [27]. The scale comprises twelve items, each rated on a seven-point Likert scale ranging from 1 (strongly disagree) to 7 (strongly agree). Total scores were calculated (range: 12–84). Higher scores on the MSPSS indicate greater perceived social support.

*Quality of life (QoL) –* The Impact of Weight on Quality of Life-Lite Questionnaire (IWQOL-Lite) was used to assess the effect of obesity on health-related quality of life on five subscales, namely physical functioning, self-esteem, sexual life, public distress, and work life. Respondents rated their level of agreement with each of the 31 items on a five-point Likert scale. Raw total scores were calculated and then transformed to a 0–100 scale (range: 0–100). Lower scores indicate a greater impact of weight on quality of life, reflecting increased challenges associated with excess body weight, whereas higher scores indicate better quality of life [28,29].

*General health –* Patients completed the 36-item Short Form Health Survey (SF-36), which is a measure of health on several health concepts: physical functioning, bodily pain, general health, social functioning, emotional wellbeing, energy/fatigue, and role limitations due to physical or emotional problems. Each item in the SF-36 is rated on a Likert-scale, with response options ranging from two to six choices. Scores for the eight health concepts are derived by transforming and summing these responses. The scores are then standardized to a scale from 0 to 100, with higher scores indicating better health and quality of life (range: 0–100) [30,31]. Table 1 provides an overview of the parameters included in the LPA.

**Table 1:** Parameters included for latent profile analysis (total or subscores).

*Eating behavior*	
- Dutch Eating Behavior Questionnaire (DEBQ)	Subscores:
	1. Emotional eating
	2. External eating
	3. Restrained eating
- Eating Disorder Examination Questionnaire (EDE-Q)	4. Total score
*Depression*	
- Beck Depression Inventory (BDI)	5. Total score
*Social support*	
- Multidimensional Scale of Perceived Social Support (MSPSS)	6. Total score
*Quality of life*	
- Impact of Weight on Quality of Life (IWQOL-Lite)	7. Total score
*General health*	
- Short-Form Health Survey (SF-36)	Subscores:
	8. Physical functioning
	9. Role limitations due to physical functioning
	10. Role limitations due to emotional problems
	11. Energy/fatigue
	12. Emotional wellbeing
	13. Social functioning
	14. Bodily pain
	15. General health

### Statistical analysis

Normality of continuous data was assessed by visual inspection of frequency histograms. Parametric continuous data were expressed as means with standard deviations (SD) and nonparametric continuous data as medians with interquartile ranges (IQR). Categorical data were expressed as frequencies with percentages.

To identify subgroups of patients based on psychosocial and behavioral indicators, LPA was applied. LPA is a model-based clustering technique that probabilistically assigns individuals to unobserved (latent) subgroups based on shared response patterns across continuous variables [32]. The goal is to detect the smallest number of distinct profiles that best represent the heterogeneity in the data, thereby capturing the most meaningful variance among participants. Fifteen parameters (i.e., the (sub)scores on the different questionnaires) were used to perform LPA (Table 1). Only participants with complete data (100% response rate) were included in the LPA. As a first step, the parameters were standardized by calculating Z-scores. Standardization was performed to ensure that variables with different scales contributed equally to the latent profile estimation, thereby preventing disproportionate influence from variables with larger scales. These Z-scores were used as input for the LPA. Subsequently, LPAs were performed to categorize subjects into groups with distinct characteristics. LPAs were estimated using the R package ‘tidyLPA’, testing models with 1–6 profiles (which is a common range used in LPA studies to balance model interpretability and statistical power [33]) across three common model structures for covariance matrices (model 1: equal variances, zero covariances; model 2: varying variances, zero covariances; model 3: equal variances and covariances). Model fit was evaluated using the Bayesian Information Criterion (BIC), Akaike Information Criterion (AIC), and entropy. Lower BIC and AIC values indicate better model fit, whereas entropy values ≥ 0.80 suggest adequate classification accuracy [34]. To ensure interpretability and model stability, each subgroup should comprise at least 5% of the population [33,35]. Following model selection, patients were assigned to the profile for which they had the highest probability of membership. Assignment certainty was assessed by examining the posterior probabilities. The best model was selected based on a combination of fit indices (BIC, AIC, entropy) and profile size. Thus, the number of profiles was not specified a priori but emerged from the data, consistent with standard LPA methodology. Stability of the selected solutions was subsequently evaluated using nonparametric bootstrapping (n = 100). In each iteration, new samples were drawn with replacement and the selected model structure was re-estimated. Stability was quantified using the Adjusted Rand Index (ARI), which compares the agreement between the bootstrapped and original profile solutions (range: 0–1), with higher ARI values indicating more consistent classification across replications [19].

Between-profile differences regarding patient characteristics and behavioral and psychosocial-based metrics were evaluated with a one-way ANOVA (normally distributed data), Kruskall-Wallis (skewed data), and Chi-square test (categorical data). For the one-way ANOVA, Tukey’s honest significant difference post-hoc test was conducted. Partial eta squared (partial η^2^) was calculated to estimate effect sizes for the ANOVA models. Pairwise group differences were quantified using Cohen’s *d*. The significance level was set at 0.05. Statistical analysis was performed using SPSS version 24 (SPSS Inc., Chicago, IL, USA).

## Results

### Sample description

Data from 272 preoperative MBS patients (77% women, n = 209) with a mean (± SD) age of 47 (± 10) years and a mean (± SD) BMI of 42 (± 5) kg/m^2^ were included in the LPA. Dyslipidemia (n = 222, 82%) and osteoarthrosis (n = 135, 50%) were the most prevalent obesity-related comorbidities. The majority had vocational education as highest education (n = 131, 48%), and most participants were married (n = 175, 64%). Patient characteristics for the total sample (n = 272) are presented in Table 2. The median time between questionnaire distribution and completion was 2 days (IQR: 0–14). The median time between questionnaire completion and surgery was 61 days (IQR: 25–101).

**Table 2:** Patient characteristics of the total population and stratified by profile.

	*Total population* *(n = 272)*	*Profile 1* *(n = 122)*	*Profile 2* *(n = 53)*	*Profile 3* *(n = 36)*	*Profile 4* *(n = 61)*	*p*
Number of patients, n (%)	272 (100.0)	122 (44.9)	53 (19.5)	36 (13.2)	61 (22.4)	NA
Age, years, mean ± SD	47.3 ± 10.1	46.2 ± 9.0	48.6 ± 10.8	45.3 ± 10.6	49.7 ± 10.8	0.069
Gender, n (%)						0.717
Female	209 (76.8)	96 (78.7)	41 (77.4)	25 (69.4)	47 (77.0)	
Male	63 (23.2)	26 (21.3)	12 (22.6)	11 (30.6)	14 (23.0)	
BMI, kg/m^2^, mean ± SD	42.4 ± 4.8	42.6 ± 4.7	43.5 ± 5.4	41.9 ± 4.4	41.2 ± 4.6	0.067
Comorbidities, n (%)						
T2DM	41 (15.1)	13 (10.7)^a^	4 (7.5)^a^	6 (16.7)^ab^	18 (29.5)^b^	0.018
Hypertension	102 (37.5)	42 (34.4)	21 (39.6)	10 (27.8)	29 (47.5)	0.198
OSAS	81 (29.8)	32 (78.7)	21 (39.6)	14 (38.9)	14 (23.0)	0.113
Osteoarthrosis	135 (49.6)	58 (47.5)	30 (56.6)	16 (44.4)	31 (50.8)	0.654
GERD	90 (33.1)	36 (29.5)	23 (43.3)	11 (30.6)	20 (32.8)	0.340
Dyslipidemia	222 (81.6)	99 (91.1)	46 (86.8)	29 (80.6)	48 (78.7)	0.720
Highest level of completed education, n (%)						0.130
No degree	7 (2.6)	2 (1.6)	0 (0.0)	2 (5.6)	3 (4.9)	
Primary school	13 (4.8)	5 (4.1)	1 (1.9)	3 (8.3)	4 (6.6)	
High school – vocational education	31 (11.4)	10 (8.2)	6 (11.3)	4 (11.1)	11 (18.0)	
High school – higher vocational education	11 (4.0)	4 (3.3)	2 (3.8)	1 (2.8)	4 (6.6)	
Vocational education	131 (48.2)	58 (47.5)	29 (54.7)	20 (55.6)	24 (39.3)	
Higher vocational education	59 (21.7)	33 (27.0)	12 (22.6)	5 (13.9)	9 (14.8)	
University	5 (1.8)	4 (3.3)	0 (0.0)	0 (0.0)	1 (1.6)	
Other	15 (5.5)	6 (4.9)	3 (5.7)	1 (2.8)	5 (8.2)	
Employment, n (%)						<0.001
Student	3 (1.1)	3 (2.5)^a^	0 (0.0)^bc^	0 (0.0)^b^	0 (0.0)^c^	
Working	183 (67.3)	96 (78.7)	33 (62.3)	26 (72.2)	28 (45.9)	
Unemployed	78 (28.7)	22 (18.0)	18 (34.0)	6 (16.7)	32 (52.5)	
Other	8 (2.9)	1 (0.8)	2 (3.8)	4 (11.1)	1 (1.6)	
Marital status, n (%)						0.026
Single	36 (13.2)	15 (12.3)^a^	4 (7.5)^a^	9 (25.0)^b^	8 (13.1)^a^	
In a relationship (not cohabiting)	8 (2.9)	3 (2.5)	1 (1.9)	3 (8.3)	1 (1.6)	
Cohabiting	35 (12.9)	16 (13.1)	12 (22.6)	0 (0.0)	7 (11.5)	
Married	175 (64.3)	81 (66.4)	35 (66.0)	18 (50.0)	41 (67.2)	
Divorced	14 (5.1)	5 (4.1)	1 (1.9)	5 (13.9)	3 (4.9)	
Widowed	4 (1.5)	2 (1.6)	0 (0.0)	1 (2.8)	1 (1.6)	
Smoking, n (%)	23 (8.5)	7 (5.7)	4 (7.5)	4 (11.1)	8 (13.1)	0.698
Alcohol consumption in units/week, n (%)						0.687
0	136 (50)	54 (44.3)	24 (45.3)	23 (63.9)	35 (57.4)	
1–7	126 (46.3)	62 (50.8)	28 (52.8)	12 (33.3)	24 (39.3)	
8–14	7 (2.6)	4 (3.3	1 (1.9)	1 (2.8)	1 (1.6)	
15–21	2 (0.7)	1 (0.8)	0 (0.0)	0 (0.0)	1 (1.6)	
> 21	1 (0.4)	1 (0.8)	0 (0.0)	0 (0.0)	0 (0.0)	

SD: Standard deviation; NA: Not applicable; BMI: Body Mass Index; T2DM: Type 2 diabetes mellitus; OSAS: Obstructive sleep apnea syndrome; GERD: Gastro-esophageal reflux disease.

^abc^Different superscript letters indicate significant differences between profiles (post-hoc Chi-square tests). Profiles sharing the same letter do not differ significantly.

### Latent profile analyses

Profile solutions were derived from the following input parameters: total scores on the BDI, EDE-Q, IWQOL-Lite, MSPSS, and subscores on the SF-36 and DEBQ (Table 1). The best-fitting LPA model was model 3 with four profiles, which showed favorable BIC (10501.1) and AIC (9841.3), and high entropy (0.967). Posterior probabilities for correct classification ranged from 0.952–0.999. Fit indices and posterior probabilities for all LPA models are presented in S1 and S2 Tables.

The selected model (model 3 with 4 profiles) showed excellent fit across bootstrap samples, as indicated by a consistently low BIC (mean = 10413, SD = 143) and AIC (mean = 9753, SD = 143), and high entropy values (mean = 0.963, SD = 0.025), supporting the model’s reliability and its ability to distinguish between latent profiles. All four profiles included more than 5% of the total sample, with subgroup sizes ranging from 36 to 122 participants (13% – 45%), thereby meeting recommended thresholds for interpretability and stability. However, classification stability across resampled datasets was moderate (mean ARI = 0.463, SD = 0.142, minimum = 0.052), suggesting that while the overall profile structure is robust, individual patient assignments may vary when the data fluctuate. In other words, the identified subgroups are stable at the group level, whereas individual-level assignments are less stable, with some patients potentially being assigned to different profiles across resamples. Bootstrap summary and density plots are presented in S3 Table and S1 Fig in the Supporting Information.

### Latent profile analyses based on behavioral and psychosocial parameters

**Fig 1:**
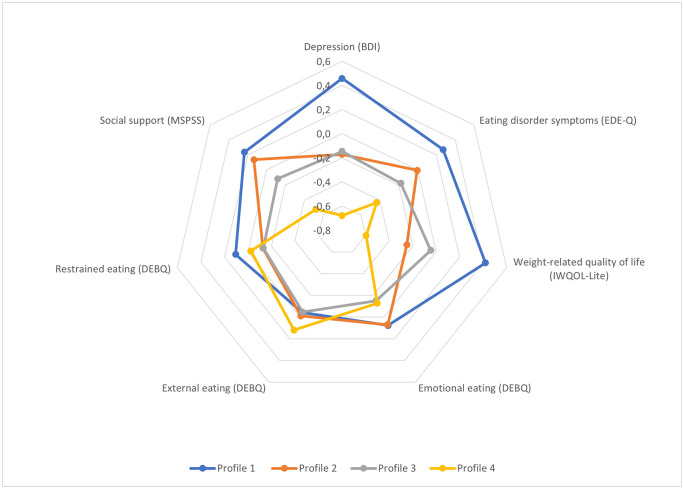
Plot of Z-scores of the behavioral and psychosocial parameters per profile. Higher scores indicate more favorable psychosocial functioning across all measures.

**Fig 2:**
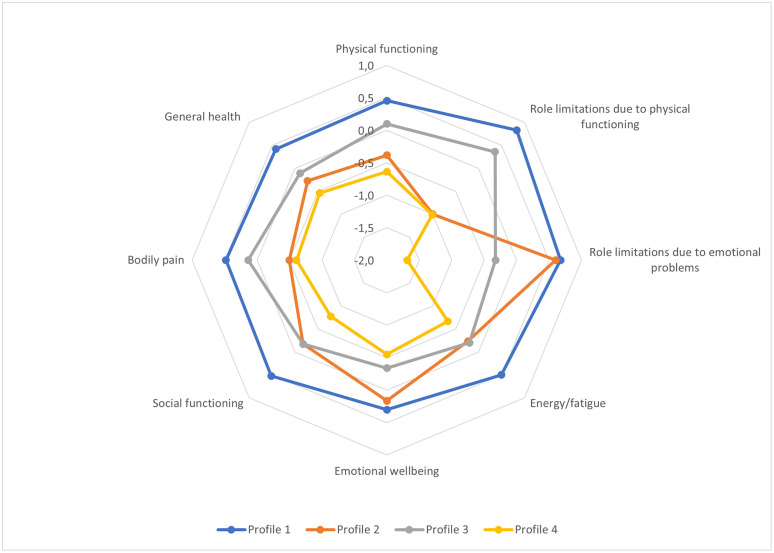
Plot of Z-scores of eight domains of the SF-36 questionnaire per profile. Higher scores indicate better health status.

LPA resulted in four profiles (profile 1: n = 122, profile 2: n = 53, profile 3: n = 36, profile 4: n = 61). The distinct behavioral and psychosocial patterns of the profiles are illustrated using standardized Z-scores across variables (Figs 1 and 2, S4 Table). Behavioral and psychosocial-based metrics of the total population and according to the profiles are presented in Table 3. The main characteristics of each profile, when compared to the others, were as follows:

**Table 3:** Behavioral and psychosocial-based metrics of the total population and stratified by profile.

	*Total population* *(n = 272)*	*Profile 1* *(n = 122)*	*Profile 2* *(n = 53)*	*Profile 3* *(n = 36)*	*Profile 4* *(n = 61)*	*P*	*Post-hoc test Tukey’s HSD*	*Partial η* ^*2*^
DEBQ, mean ± SD								
Emotional eating	2.1 ± 0.7	2.1 ± 0.7	2.1 ± 0.7	2.2 ± 0.8	2.2 ± 0.8	0.428	NA	NA
External eating	2.6 ± 0.6	2.6 ± 0.6	2.6 ± 0.6	2.6 ± 0.7	2.5 ± 0.7	0.763	NA	NA
Restrained eating	3.0 ± 0.6	2.9 ± 0.7	3.1 ± 0.5	3.1 ± 0.7	3.0 ± 0.7	0.415	NA	NA
EDE-Q, mean ± SD	2.1 ± 0.9	1.8 ± 0.9	2.1 ± 0.6	2.2 ± 1.0	2.5 ± 1.1	<0.001	4 > 1	0.079
BDI, mean ± SD	8.6 ± 5.7	6.0 ± 4.0	9.6 ± 5.0	9.4 ± 6.1	12.5 ± 6.3	<0.001	4 > 2, 3 > 1	0.206
MSPSS, mean ± SD	71.0 ± 13.8	74.2 ± 11.0	72.8 ± 10.1	69.4 ± 11.7	63.8 ± 19.2	<0.001	1, 2 > 4	0.092
IWQOL-Lite, mean ± SD	62.3 ± 18.0	69.8 ± 14.6	57.8 ± 13.6	61.5 ± 20.9	51.6 ± 19.1	<0.001	1 > 2, 3, 4 3 > 4	0.172
SF-36, mean ± SD								
Physical functioning	58.8 ± 21.7	68.7 ± 16.5	50.5 ± 19.1	61.0 ± 21.7	45.0 ± 23.0	<0.001	1, 3 > 2, 4	0.215
Role limitations due to physical functioning	55.7 ± 40.2	89.1 ± 16.7	15.6 ± 20.3	70.1 ± 25.2	15.2 ± 22.9	<0.001	1 > 3 > 2, 4	0.751
Role limitations due to emotional problems	76.8 ± 34.2	100.0 ± 0.0	97.5 ± 8.9	65.7 ± 5.6	19.1 ± 16.6	<0.001	1, 2 > 3 > 4	0.931
Energy/fatigue	53.1 ± 18.8	62.4 ± 15.0	48.7 ± 15.9	49.3 ± 18.9	40.5 ± 18.	<0.001	1 > 2, 3 > 4	0.228
Emotional wellbeing	69.6 ± 9.0	72.3 ± 6.6	71.1 ± 6.7	66.6 ± 10.5	64.7 ± 11.2	<0.001	1, 2 > 3, 4	0.130
Social functioning	77.0 ± 23.4	89.1 ± 14.5	72.9 ± 24.5	72.9 ± 24.2	58.8 ± 22.7	<0.001	1 > 2, 3 > 4	0.268
Bodily pain	58.1 ± 24.3	69.8 ± 19.6	46. 1 ± 20.7	61.5 ± 24.7	43.4 ± 23.0	<0.001	1, 3 > 2, 4	0.238
General health	48.1 ± 18.8	56.0 ± 18.2	43.0 ± 16.7	46.1 ± 17.5	38.0 ± 15.9	<0.001	1 > 2, 3, 4	0.160

DEBQ: Dutch eating behavior questionnaire; SD: Standard deviation; EDE-Q: Eating disorder examination questionnaire; BDI: Beck depression inventory; MSPSS: Multidimensional scale of perceived social support; IWQOL-Lite: Impact of weight on quality of life questionnaire; SF-36: Short form health survey.

- **Profile 1**, the *‘higher psychosocial and physical functioning profile’* (n = 122, 45%) was characterized by the highest scores on all domains of the SF-36, IWQOL-Lite, and MSPSS questionnaires, indicating the highest levels of quality of life and perceived social support. Moreover, this profile demonstrated the lowest levels of depressive symptoms, restrained eating, emotional eating, and disordered eating behavior.
- **Profile 2,** the *‘higher psychosocial and lower physical functioning profile’* comprised 20% (n = 53) of the sample and was characterized by moderate depression, strong emotional well-being and social support, and few limitations due to emotional problems. However, this profile faced physical challenges, including low physical functioning, bodily pain, and role limitations due to physical functioning.
- **Profile 3,** the *‘lower psychosocial functioning and stable physical health profile’* was the smallest group (n = 36, 13%). Patients in this profile reported moderate to good general health with few physical limitations. However, they experienced lower emotional well-being and difficulties with daily activities due to emotional problems. Depressive symptoms and disordered eating behavior were present at moderate levels.
- **Profile 4,** the *‘lower psychosocial and physical functioning profile’* encompassed 22% (n = 61) of the sample. Patients in this profile exhibited the highest levels of depression, external eating, and disordered eating behavior. Additionally, they reported the lowest levels of quality of life, perceived social support, and general health.

Examination of the z-scores revealed significant differences between profiles on the EDE-Q, BDI, MSPSS, IWQOL-Lite, and all SF-36 subscales, except for the DEBQ (Table 3). Despite these significant z-score differences, some parameters showed limited variation in absolute scores. For example, most profiles were characterized by uniformly high scores on the MSPSS and low scores on the BDI, indicating minimal variation in these measures in absolute terms (Table 3). This contrasts with the quality-of-life domains (SF-36 and IWQOL-Lite), which exhibited greater variability. In particular, absolute scores on the IWQOL-Lite and several SF-36 subscales, specifically ‘role limitations due to physical functioning’, ‘role limitations due to emotional problems’, and ‘social functioning’ varied more substantially between profiles. These differences are clinically meaningful, as absolute scores – rather than z-scores – are relied upon in clinical practice.

To further characterize differences between profiles, partial η^2^ values were calculated to quantify the overall proportion of variance in each indicator explained by profile membership (Table 3). The largest effects were observed for role limitations due to emotional problems (partial η^2^ = 0.931) and role limitations due to physical functioning (partial η^2^ = 0.751), indicating that these domains contributed most strongly to profile differentiation. Pairwise differences between the four latent profiles were subsequently examined using pairwise Cohen’s *d* for each standardized indicator. The largest effect sizes were observed for role limitations due to emotional problems (profile 1 vs 3; *d* = 13.02), role limitations due to physical functioning (profile 1 vs 2; *d* = 4.12), and social functioning (profile 1 vs 4; *d* = 1.71), all indicating large differences between profiles. A complete effect size matrix for all indicators and all profile comparisons is provided in S5 Table.

### Between-profile differences

Baseline characteristics by profile are shown in Table 2. Statistically significant between-profile-differences were observed for employment status, marital status, and the presence of type 2 diabetes mellitus (T2DM). T2DM was most prevalent in profile 4 (the ‘*lower psychosocial and physical functioning profile’*), where nearly one-third of patients (30%, n = 18) had this comorbidity, compared to 11% in profile 1 (n = 13), 8% in profile 2 (n = 4), and 17% in profile 3 (n = 6). Regarding employment, students were exclusively observed in profile 1 (n = 3, 3%). Individuals in paid employment were most represented in profiles 1 (79%, n = 96) and 3 (72%, n = 26). In contrast, unemployment was most prevalent in profile 4, with more than half of the participants (53%, n = 32) being unemployed, followed by 34% in profile 2 (n = 18), 18% in profile 1 (n = 22), and 17% in profile 3 (n = 6). Differences in marital status also merged. The proportion of married individuals was lowest in profile 3 (*‘lower psychosocial functioning and stable physical health profile’*) at 50%, compared to approximately two-thirds in the other profiles (66–67%). This lower marriage rate in profile 3 corresponded with higher proportions of single (25%, n = 9) and divorced (14%, n = 5) individuals. Furthermore, none of the participants in profile 3 reported cohabiting, in contrast to 23% in profile 2, 13% in profile 1, and 12% in profile 4. There were no statistically significant differences in age, gender, BMI, level of education, smoking, and alcohol consumption between the profiles.

Because the inclusion period (July 2019 – December 2022) overlapped with the COVID-19 pandemic, a sensitivity analysis was conducted in which participants were stratified into three groups based on time of inclusion (pre-COVID, during COVID, and post-COVID). No significant differences were observed between groups for most demographic, behavioral, and psychosocial variables. The only exception was the IWQOL score, which was higher in the post-COVID group compared to the COVID group (S1 Appendix, S6-S8 Tables).

## Discussion

### Main findings

This study applied LPA to identify subgroups in patients undergoing MBS based on behavioral and psychosocial characteristics, and to examine differences in baseline characteristics. Four distinct profiles were identified, with most behavioral and psychosocial variables showing significant differences between profiles. These profiles illustrate the heterogeneity in psychosocial and behavioral functioning among patients seeking MBS and may provide a conceptual framework for understanding different care needs when initially seeking treatment. In theory, such heterogeneity can be interpreted as reflecting different positions along a continuum of behavioral and psychosocial vulnerability, encompassing variations in emotional wellbeing, depressive symptoms, and problematic eating behaviors. This perspective could justify differentiated supportive care. Previous profiling in patients undergoing MBS has focused on relatively stable personality traits [7–9], whereas our study considers a broader set of modifiable psychosocial and behavioral factors. This approach may be more actionable for preoperative interventions by targeting changeable barriers rather than fixed traits. For example, profile 1 (‘*higher psychosocial and physical functioning profile*’) might find the standard care pathway unnecessarily intensive, whereas those in profile 4 (‘*lower psychosocial and physical functioning profile*’) are likely to benefit from additional support across multiple domains [36]. Profile 2, identified as ‘*higher psychosocial and lower physical functioning profile*’, may benefit from physiotherapy to enhance physical functioning, reduce role limitations due to physical functioning, and alleviate bodily pain [37,38]. In contrast, profile 3 (*‘lower psychosocial functioning and stable physical health profile’*) could benefit from additional support from a dietitian or psychologist to address problematic eating behaviors or emotional wellbeing [14,39]. Importantly, these examples are intended to illustrate potential directions for tailored care and are not based on evidence that assigning interventions according to profile membership improves outcomes.

Before these profiles can be translated into a clinically applicable prognostic model, further refinement is required. In the present study, patient assignment to profiles was based on probabilistic estimation rather than deterministic classification. Given that the stability of classifications across resampled datasets was only moderate (ARI = 0.463), individual profile assignments may vary with fluctuations in the data. Consequently, some patients might be allocated to different profiles across resamples. Therefore, the profiles in this study are not suitable for guiding personalized treatment at the individual level. To advance toward a clinically applicable prognostic model, future research should aim to identify discriminative variables that consistently distinguish between profiles and enable accurate patient classification. In this context, the current findings already suggest that certain measures, such as the IWQOL-Lite total score and specific SF-36 subscales, possess greater discriminative value between profiles than others, including the DEBQ subscales. In contrast, MSPSS scores consistently indicated high perceived support across profiles, and BDI scores reflected minimal or borderline depressive symptoms, suggesting that these variables contribute less to profile differentiation. Taken together, these insights can guide the selection of key variables for future prognostic modelling. Furthermore, establishing empirically derived cutoff values may further support this process by enabling more precise allocation of individual patients to specific profiles. Such classification represents an essential step toward developing targeted interventions and personalized treatment pathways. Although not yet ready for clinical implementation, the identified profiles clearly reveal distinct preoperative MBS subgroups with differing behavioral and psychosocial characteristics, highlighting the potential for a more tailored clinical approach.

Variability in psychosocial and behavioral functioning across profiles may help explain differences in long-term treatment outcomes. While most demographic and clinical variables (e.g., age, gender, BMI, obesity-related comorbidities (except T2DM), smoking behavior) did not differ significantly between profiles, several psychosocially relevant baseline characteristics did. This suggests that relevant heterogeneity lies primarily in behavioral and psychosocial factors rather than in standard clinical measures, highlighting the importance of assessing these factors in clinical practice to determine the care requirements of different patient profiles. Prior research in MBS populations has largely focused on isolated predictors of postoperative outcomes. Although mental health, composite psychological functioning, and binge eating have been associated with postoperative weight loss outcomes [11,12,15–17], the evidence remains mixed, and the impact of lifestyle and psychosocial factors is not unequivocal. This inconsistency may reflect the limited explanatory power of single-variable approaches. While no conclusions can be drawn regarding postoperative outcomes, the present profile-based approach offers a more integrative perspective by capturing the co-occurrence and interaction of multiple psychosocial and behavioral factors. This aligns with the biopsychosocial model, which conceptualizes health outcomes as the result of dynamic interactions between psychological, behavioral, and biological domains. Moreover, not only the presence but also the severity of psychological symptoms appears relevant [16]. From this perspective, the identified profiles may represent distinct configurations along a continuum of psychosocial vulnerability, including variations in emotional wellbeing, depressive symptoms, and problematic eating behaviors.

Nevertheless, a few baseline characteristics did differ between profiles, namely employment status, the presence of the comorbidity T2DM, and marital status. Profile 2 (‘*higher psychosocial and lower physical functioning profile*’) and profile 4 (‘*lower psychosocial and physical functioning profile’*) exhibited higher rates of unemployment compared to profile 1 (‘*higher psychosocial and physical functioning profile’*) and profile 3 (‘*lower psychosocial functioning and stable physical health profile’*). Interestingly, these differences were not explained by education level, suggesting that poor physical health – reflected in low physical functioning, bodily pain, and role limitations – could potentially contribute to workforce exclusion. However, due to the cross-sectional design of this study, no conclusions can be drawn regarding causality. Future research is needed to determine whether improvements in physical functioning, reductions in bodily pain, and lessen role limitations following MBS are associated with an increased likelihood of returning to employment.

Differences in physical health were also reflected in the prevalence of T2DM. Higher rates of T2DM were observed in profile 4 (‘*lower psychosocial and physical functioning profile’*), compared to profile 1 and 2. This could potentially explain the lower health-related QoL observed in profile 4, as the presence of T2DM is known to negatively affect health-related QoL [40,41]. Similarly, profile 3 *(‘lower psychosocial functioning and stable physical health profile’)* also showed a higher prevalence of T2DM compared to profiles 1 and 2. In this profile, higher scores for external eating, emotional eating, and disordered eating behavior were observed. These findings support previous literature indicating that, in individuals with T2DM, the presence of disordered eating is associated with poorer glycemic control, particularly in the context of higher BMI [42]. Therefore, addressing problematic eating behavior within this profile may be relevant in clinical practice and could potentially contribute to improved diabetes regulation.

Marital status mirrored variations in emotional wellbeing. A smaller proportion of participants in profile 3 were married (*‘lower psychosocial functioning and stable physical health profile’*) compared to those in profiles 1, 2, and 4, while the proportions of single and divorced individuals were higher. These relationship patterns could potentially explain the lower levels of emotional wellbeing observed in this group, as being in a supportive relationship has been associated with improved mental health [43]. Conversely, reduced emotional wellbeing may also decrease the likelihood of forming or maintaining a relationship.

Psychosocial profiling may be valuable to understand patient vulnerability and guide targeted support. Behavioral and psychosocial profiling can also provide patients with insights into their own characteristics across various domains in comparison with other individuals undergoing MBS. Profiling may therefore help patients recognize areas for lifestyle improvement and increase awareness of personal challenges and vulnerabilities, which in turn could motivate patients to seek additional therapy, support, or targeted interventions. Furthermore, profiling might help manage patient expectations and reduce postoperative disappointment, but research is needed first to determine whether postoperative outcomes, such as weight loss and the improvement of obesity-related comorbidities, differ between profiles.

Despite the potential of behavioral and psychosocial profiling to improve understanding of patient heterogeneity, it also raises important ethical considerations in clinical practice. Labeling patients carries a risk of stigmatization or clinician bias, particularly if labels are interpreted as judgments about a patient’s motivation, capabilities, or suitability for surgery. Profiles are intended as supportive tools to help clinicians identify areas where patients may benefit from additional resources or guidance, rather than as criteria to limit access to care or reduce standard support. Communication of profile information should be transparent and patient-centered, ensuring that patients understand the purpose of profiling and how it may help optimize support. Ethical application requires that profiling be used to enhance care quality and patient experience, rather than serving as a basis for punitive or exclusionary decision-making.

### Strengths and limitations

A strength of our study is the identification of distinct psychosocial and behavioral subgroups in the population undergoing MBS using LPA in a prospective study sample, focusing on potentially modifiable factors and capturing a broad range of behavioral and psychosocial characteristics. This approach complements previous research, which has often focused on more unidimensional and less readily modifiable factors. The primary limitation of this study is the potential for selection bias, as individuals who chose to participate may not be fully representative of the broader population of patients prior to MBS. Unfortunately, data on individuals who declined to participate were not available, limiting the ability to assess potential differences between participants and non-participants. Second, generalizability of the findings is limited. The sample was drawn from a single Dutch bariatric center and consisted predominantly of women, which may restrict generalizability to broader MBS populations. Furthermore, exclusion of individuals with insufficient Dutch language proficiency may have reduced representation of immigrant and minority groups. Compared with large multicenter cohorts such as the Longitudinal Assessment of Bariatric Surgery (LABS) study in the United States (US), our cohort showed a similar proportion of female participants and a predominantly white ethnic composition [44]. However, differences were observed in age, baseline BMI, and comorbidity profiles: participants in the LABS cohort tended to be younger and presented with higher BMI. Moreover, T2DM and hypertension were more prevalent in the LABS study, while dyslipidemia was more common in our cohort (S9 Table). These demographic differences, together with variation in healthcare systems and cultural context, may influence both the composition of psychosocial profiles and their feasibility in clinical practice. The Dutch healthcare system is characterized by universal coverage, strong primary care gatekeeping, and structured multidisciplinary bariatric care pathways, whereas the US system is more fragmented, with greater heterogeneity in insurance coverage, referral pathways, and access to specialist care. These structural differences may affect which patients are evaluated for MBS and at what stage of disease severity, potentially influencing distribution and composition of preoperative lifestyle profiles. Therefore, replication and validation in diverse clinical settings and populations are needed to determine whether the identified profiles are reproducible beyond the present context. Third, the inclusion period overlapped with the COVID-19 pandemic, which may have influenced psychosocial wellbeing and quality of life. Although IWQOL-Lite scores were higher in the post-COVID group compared with the COVID group, the absolute mean difference was small (5.7 points on a 0–100 scale), suggesting limited clinical relevance. Therefore, pandemic-related effects are unlikely to have substantially influenced the identified profiles. Lastly, caution is warranted when applying the profiles for individual-level clinical decision-making, as discussed earlier. Given the moderate classification stability across resampled datasets (ARI = 0.463), the profiles in this study are not yet suitable for guiding personalized treatment at the individual level. Further steps are needed to advance toward this goal. These include the identification of variables with high discriminative power, the establishment of derived cutoff values to enable reliable patient classification, and the examination of whether profile membership predicts long-term clinical outcomes. Improving classification performance will require the use of larger and more diverse samples, the inclusion of additional discriminative factors, and the exploration of alternative model specifications, including different covariance structures.

### Future perspectives

The potential of the profiles to support routine decision-making in MBS care should be evaluated. Longitudinal follow-up studies are required to determine whether the identified preoperative profiles have prognostic validity for postoperative treatment outcomes such as weight loss, resolution of obesity-related comorbidities, other health-related outcomes, and overall well-being after MBS. These findings could support the development of tailored treatment strategies to specific patient subgroups, where the profiles are intended as supportive tools, helping clinicians to identify areas where patients may benefit from additional guidance or resources, rather than as criteria to limit access to care or reduce standard support. In addition, future research should examine how the identified profiles evolve over time to determine which types of support may be most effective at each stage. For example, patients in profile 2 (‘*higher psychosocial and lower physical functioning profile’*) may experience physical improvements following weight loss, potentially reducing the need for physiotherapy. As a result, therapeutic focus may shift toward other domains, such as emotional or psychosocial functioning, which may become more prominent over time. Understanding these transitions could help tailor interventions more precisely to the changing’s needs of each subgroup. Such insights could also identify the optimal assessment point for evaluation to detect and treat high-risk patients as early as possible. Replication of the identified subgroups in other populations before MBS is needed to enhance generalizability. Lastly, future efforts should focus on enabling the application of the profiles at the individual level, which may be facilitated by the steps outlined above.

## Conclusion

Our study identified profiles based on behavioral and psychosocial parameters in patients prior to MBS. Four distinct profiles emerged, which may be useful for guiding profile-based treatment regimens. More research is needed to ascertain whether the profiles are useful in determining who truly benefits from MBS and to apply the profiles at the individual level.

## Supporting information

S1 Appendix Sensitivity analyses: stratification by COVID-19 period. (DOCX)

S1 Fig Density plots. (DOCX)

S1 Table Fit indices for latent profile analyses. (DOCX)

S2 Table Posterior probabilities. (DOCX)

S3 Table Bootstrap results for each run. (DOCX)

S4 Table Mean values per indicator variable by profile. (DOCX)

S5 Table Effect size matrix. (DOCX)

S6 Table Distribution of patients per profile by COVID-19 period. (DOCX)

S7 Table Patient characteristics of the total population and stratified by COVID-19 period. (DOCX)

S8 Table Behavioral and psychosocial-based metrics of the total population and stratified by COVID-19 period. (DOCX)

S9 Table Baseline characteristics of the study population and the LABS cohort. (DOCX)

## References

[1] DATO jaarverslag 2023. 2023. https://dica.nl/wp-content/uploads/2024/07/DATO-jaarverslag-2023.pdf

[2] Colquitt JL, Pickett K, Loveman E, Frampton GK. Surgery for weight loss in adults. Cochrane Database Syst Rev. 2014;2014(8):CD003641. doi: 10.1002/14651858.CD003641.pub4 25105982 PMC9028049

[3] Karmali S, Brar B, Shi X, Sharma AM, de Gara C, Birch DW. Weight recidivism post-bariatric surgery: A systematic review. Obes Surg. 2013;23(11):1922–33. doi: 10.1007/s11695-013-1070-4 23996349

[4] Fischer L, Nickel F, Sander J, Ernst A, Bruckner T, Herbig B, et al. Patient expectations of bariatric surgery are gender specific--a prospective, multicenter cohort study. Surg Obes Relat Dis. 2014;10(3):516–23. doi: 10.1016/j.soard.2014.02.040 24951069

[5] Ahlich E, Verzijl CL, Cunning A, Wright E, Rancourt D. Patient motivations and goals for bariatric surgery: A mixed methods study. Surg Obes Relat Dis. 2021;17(9):1591–602. doi: 10.1016/j.soard.2021.05.017 34134941

[6] van der Laan L, Kuipers EAM, Timmerman JG, Kaijser MA, van Det MJ, Emous M. Patient expectations of bariatric outcomes, baseline, and long-term evaluation. Obes Surg. 2025;35(8):3064–74. doi: 10.1007/s11695-025-07997-0 40614005 PMC12380636

[7] Claes L, Vandereycken W, Vandeputte A, Braet C. Personality subtypes in female pre-bariatric obese patients: do they differ in eating disorder symptoms, psychological complaints and coping behaviour?. Eur Eat Disord Rev. 2013;21(1):72–7. doi: 10.1002/erv.2188 22807095

[8] Müller A, Claes L, Wilderjans TF, de Zwaan M. Temperament subtypes in treatment seeking obese individuals: A latent profile analysis. Eur Eat Disord Rev. 2014;22(4):260–6. doi: 10.1002/erv.2294 24809764

[9] Schäfer L, Hübner C, Carus T, Herbig B, Seyfried F, Kaiser S, et al. Identifying prebariatric subtypes based on temperament traits, emotion dysregulation, and disinhibited eating: A latent profile analysis. Int J Eat Disord. 2017;50(10):1172–82. doi: 10.1002/eat.22760 28815744

[10] Livhits M, Mercado C, Yermilov I, Parikh JA, Dutson E, Mehran A, et al. Exercise following bariatric surgery: systematic review. Obes Surg. 2010;20(5):657–65. doi: 10.1007/s11695-010-0096-0 20180039 PMC2850994

[11] Monpellier VM, Janssen IMC, Antoniou EE, Jansen ATM. Weight change after Roux-en Y gastric bypass, physical activity and eating style: Is there a relationship?. Obes Surg. 2019;29(2):526–33. doi: 10.1007/s11695-018-3560-x 30392103

[12] Colles SL, Dixon JB, O’Brien PE. Grazing and loss of control related to eating: two high-risk factors following bariatric surgery. Obesity (Silver Spring). 2008;16(3):615–22. doi: 10.1038/oby.2007.101 18239603

[13] Amundsen T, Strømmen M, Martins C. Suboptimal Weight loss and weight regain after gastric bypass surgery-postoperative status of energy intake, eating behavior, physical activity, and psychometrics. Obes Surg. 2017;27(5):1316–23. doi: 10.1007/s11695-016-2475-7 27914028 PMC5403843

[14] Miller-Matero LR, Bryce K, Saulino CK, Dykhuis KE, Genaw J, Carlin AM. Problematic eating behaviors predict outcomes after bariatric surgery. Obes Surg. 2018;28(7):1910–5. doi: 10.1007/s11695-018-3124-0 29417489

[15] Romeijn MM, Schellekens J, Bonouvrie DS, Janssen L, van Dielen FMH, Leclercq WKG, et al. Emotional eating as predictor of weight loss 2 years after Roux-en-Y gastric bypass. Clin Obes. 2021;11(4):e12458. doi: 10.1111/cob.12458 34053188 PMC8365652

[16] Wimmelmann CL, Dela F, Mortensen EL. Psychological predictors of weight loss after bariatric surgery: A review of the recent research. Obes Res Clin Pract. 2014;8(4):e299-313. doi: 10.1016/j.orcp.2013.09.003 25091351

[17] Vermeer KJ, Monpellier VM, Cahn W, Janssen IMC. Bariatric surgery in patients with psychiatric comorbidity: Significant weight loss and improvement of physical quality of life. Clin Obes. 2020;10(4):e12373. doi: 10.1111/cob.12373 32424972 PMC9285938

[18] Conceição EM, Fernandes M, de Lourdes M, Pinto-Bastos A, Vaz AR, Ramalho S. Perceived social support before and after bariatric surgery: Association with depression, problematic eating behaviors, and weight outcomes. Eat Weight Disord. 2020;25(3):679–92. doi: 10.1007/s40519-019-00671-2 30859467

[19] Dalmaijer ES, Nord CL, Astle DE. Statistical power for cluster analysis. BMC Bioinformatics. 2022;23(1):205. doi: 10.1186/s12859-022-04675-1 35641905 PMC9158113

[20] Eichler J, Schmidt R, Bartl C, Benecke C, Strauss B, Brähler E, et al. Self-regulation profiles reflecting distinct levels of eating disorder and comorbid psychopathology in the adult population: A latent profile analysis. Int J Eat Disord. 2023;56(2):418–27. doi: 10.1002/eat.23857 36420839

[21] Maltais-Lévesque C, Legendre M, Bégin C. Examining maladaptive eating behaviors and psychological difficulties among women with compulsive eating and obesity: A latent profile analysis. J Eat Disord. 2025;13(1):36. doi: 10.1186/s40337-025-01193-2 39972394 PMC11841286

[22] Dai Y, Zheng Y, Hu K, Chen J, Lu S, Li Q, et al. Heterogeneity in the co-occurrence of depression and anxiety among adolescents: Results of latent profile analysis. J Affect Disord. 2024;357:77–84. doi: 10.1016/j.jad.2024.04.065 38670464

[23] Fried M, Yumuk V, Oppert JM, Scopinaro N, Torres A, Weiner R. Interdisciplinary European guidelines on metabolic and bariatric surgery. Obesity Surgery. 2014;24(1):42–55. doi: 10.1007/s11695-013-1079-8 24081459

[24] Van Strien T, Frijters JE, Bergers GP, Defares PB. The Dutch Eating Behavior Questionnaire (DEBQ) for assessment of restrained, emotional, and external eating behavior. Int J Eat Disord. 1986;5(2):295–315. doi: 10.1002/1098-108X(198602)5:2<295::AID-EAT2260050209>3.0.CO;2-T

[25] Fairburn CG, Beglin SJ. Assessment of eating disorders: Interview or self-report questionnaire?. Int J Eat Disord. 1994;16(4):363–70. doi: 10.1002/1098-108x(199412)16:4<363::aid-eat2260160405>3.0.co;2-# 7866415

[26] Beck AT, Steer RA, Brown GK. Beck Depression Inventory-II Manual. Psychological Corporation. 1996. doi: 10.1037/t00742-000

[27] Zimet GD, Powell SS, Farley GK, Werkman S, Berkoff KA. Psychometric characteristics of the Multidimensional Scale of Perceived Social Support. J Pers Assess. 1990;55(3–4):610–7. doi: 10.1080/00223891.1990.9674095 2280326

[28] Kolotkin RL, Crosby RD, Kosloski KD, Williams GR. Development of a brief measure to assess quality of life in obesity. Obes Res. 2001;9(2):102–11. doi: 10.1038/oby.2001.13 11316344

[29] Kolotkin RL, Crosby RD, Williams GR. Health-related quality of life varies among obese subgroups. Obes Res. 2002;10(8):748–56. doi: 10.1038/oby.2002.102 12181383

[30] Aaronson NK, Muller M, Cohen PD, Essink-Bot ML, Fekkes M, Sanderman R, et al. Translation, validation, and norming of the Dutch language version of the SF-36 Health Survey in community and chronic disease populations. J Clin Epidemiol. 1998;51(11):1055–68. doi: 10.1016/s0895-4356(98)00097-3 9817123

[31] 36-Item Short Form Health Survey. SF-36. https://meetinstrumentenzorg.nl/wp-content/uploads/instrumenten/SF-36-RAND-36-meetinstr.pdf

[32] Jansen M, Bakker MM, Sepriano AR, Shkedy Z, Landewé RL, Ramiro S, et al. Use of clustering techniques for clinical and epidemiological research: practical tips using an example from rheumatology. J Clin Epidemiol. 2026;192:112183. doi: 10.1016/j.jclinepi.2026.112183 41679518

[33] Tein J-Y, Coxe S, Cham H. Statistical power to detect the correct number of classes in latent profile analysis. Struct Equ Modeling. 2013;20(4):640–57. doi: 10.1080/10705511.2013.824781 24489457 PMC3904803

[34] Clark SL, Muthén B. Relating latent class analysis results to variables not included in the analysis. https://www.statmodel.com/download/relatinglca.pdf. 2009.

[35] Ferguson SL, Moore GEW, Hull DM. Finding latent groups in observed data: A primer on latent profile analysis in Mplus for applied researchers. Int J Behav Dev. 2020;44(5):458–68. doi: 10.1177/0165025419881721

[36] Lau T, Schild S, Klos B, Schraml J, Archid R, Stengel A, et al. Psychological benefits of a preoperative educational bridging program for bariatric surgery: Does face-to-face versus videoconference-based delivery make a difference?. Obes Facts. 2024;17(6):553–69. doi: 10.1159/000539797 39019026 PMC11661839

[37] Schumacher LM, Wu Y, Thomas JG, Baillot A, Papasavas PK, Vithiananthan S, et al. Preoperative 24-hour movement behaviors and early weight loss after metabolic bariatric surgery: A compositional analysis. Int J Obes (Lond). 2026;50(3):679–83. doi: 10.1038/s41366-025-01983-3 41381935 PMC12704823

[38] Bond DS, Thomas JG, Vithiananthan S, Unick J, Webster J, Roye GD, et al. Intervention-related increases in preoperative physical activity are maintained 6-months after Bariatric surgery: Results from the bari-active trial. Int J Obes (Lond). 2017;41(3):467–70. doi: 10.1038/ijo.2016.237 28025574 PMC5340609

[39] Paul L, van der Heiden C, van Hoeken D, Deen M, Vlijm A, Klaassen RA, et al. Cognitive behavioral therapy versus usual care before bariatric surgery: One-year follow-up results of a randomized controlled trial. Obes Surg. 2021;31(3):970–9. doi: 10.1007/s11695-020-05081-3 33170444 PMC7921027

[40] Janssen LMM, Hiligsmann M, Elissen AMJ, Joore MA, Schaper NC, Bosma JHA, et al. Burden of disease of type 2 diabetes mellitus: cost of illness and quality of life estimated using the Maastricht Study. Diabet Med. 2020;37(10):1759–65. doi: 10.1111/dme.14285 32112462 PMC7539911

[41] Leal J, Becker F, Feenstra T, Pagano E, Jensen TM, Vistisen D, et al. Health-related quality of life for normal glycaemia, prediabetes and type 2 diabetes mellitus: Cross-sectional analysis of the ADDITION-PRO study. Diabet Med. 2022;39(6):e14825. doi: 10.1111/dme.14825 35253278 PMC9311436

[42] Papelbaum M, de Oliveira Moreira R, Coutinho WF, Kupfer R, Freitas S, Raggio Luz R, et al. Does binge-eating matter for glycemic control in type 2 diabetes patients?. J Eat Disord. 2019;7:30. doi: 10.1186/s40337-019-0260-4 31516703 PMC6728934

[43] Holt-Lunstad J, Birmingham W, Jones BQ. Is there something unique about marriage? The relative impact of marital status, relationship quality, and network social support on ambulatory blood pressure and mental health. Ann Behav Med. 2008;35(2):239–44. doi: 10.1007/s12160-008-9018-y 18347896

[44] Courcoulas AP, King WC, Belle SH, Berk P, Flum DR, Garcia L, et al. Seven-year weight trajectories and health outcomes in the longitudinal assessment of bariatric surgery (LABS) study. JAMA Surg. 2018;153(5):427–34. doi: 10.1001/jamasurg.2017.5025 29214306 PMC6584318

