# Supplementary material for: Identifying subgroups of individuals undergoing metabolic bariatric surgery based on behavioral and psychosocial factors: A latent profile analysis
Source: PLoS One. 2026 Jun 24;21(6):e0352252. doi: 10.1371/journal.pone.0352252 (PMC13293419; doi:10.1371/journal.pone.0352252)
Supplement: S1 Appendix — (DOCX) [file pone.0352252.s001.docx]

**S1 Appendix. Sensitivity analyses: stratification by COVID-19 period**

Given that the inclusion period (July 2019 - December 2022) overlapped with the COVID-19 pandemic, we performed an additional sensitivity analysis in which participants were stratified according to the timing of inclusion relative to the pandemic. Three time periods were defined:

1. Pre-COVID period: inclusion before March 2020.
2. COVID period: inclusion between March 2020 and January 2022, during which national lockdown measures and healthcare restrictions were in place in the Netherlands.
3. Post-COVID period: inclusion from February 2022 onwards, when most restrictions had been lifted.

The distribution of patients across the different COVID-19 periods was assessed (Table S7). Parametric continuous data were expressed as means with standard deviations (SD) and nonparametric continuous data as medians with interquartile ranges (IQR). Categorical data were expressed as frequencies with percentages. For normally distributed data, a one-way ANOVA with Tukey’s honest significant difference post-hoc test was used to assess differences in patient characteristics and behavioral and psychosocial metrics across groups defined by time period (pre-COVID, COVID, and post-COVID). For skewed data, a Kruskal-Wallis test was performed. The results are presented in Table S8.
