# Supplementary material for: Identifying subgroups of individuals undergoing metabolic bariatric surgery based on behavioral and psychosocial factors: A latent profile analysis
Source: PLoS One. 2026 Jun 24;21(6):e0352252. doi: 10.1371/journal.pone.0352252 (PMC13293419; doi:10.1371/journal.pone.0352252)
Supplement: S1 Fig — (DOCX) [file pone.0352252.s002.docx]

**S1 Figure. Density plots**

**
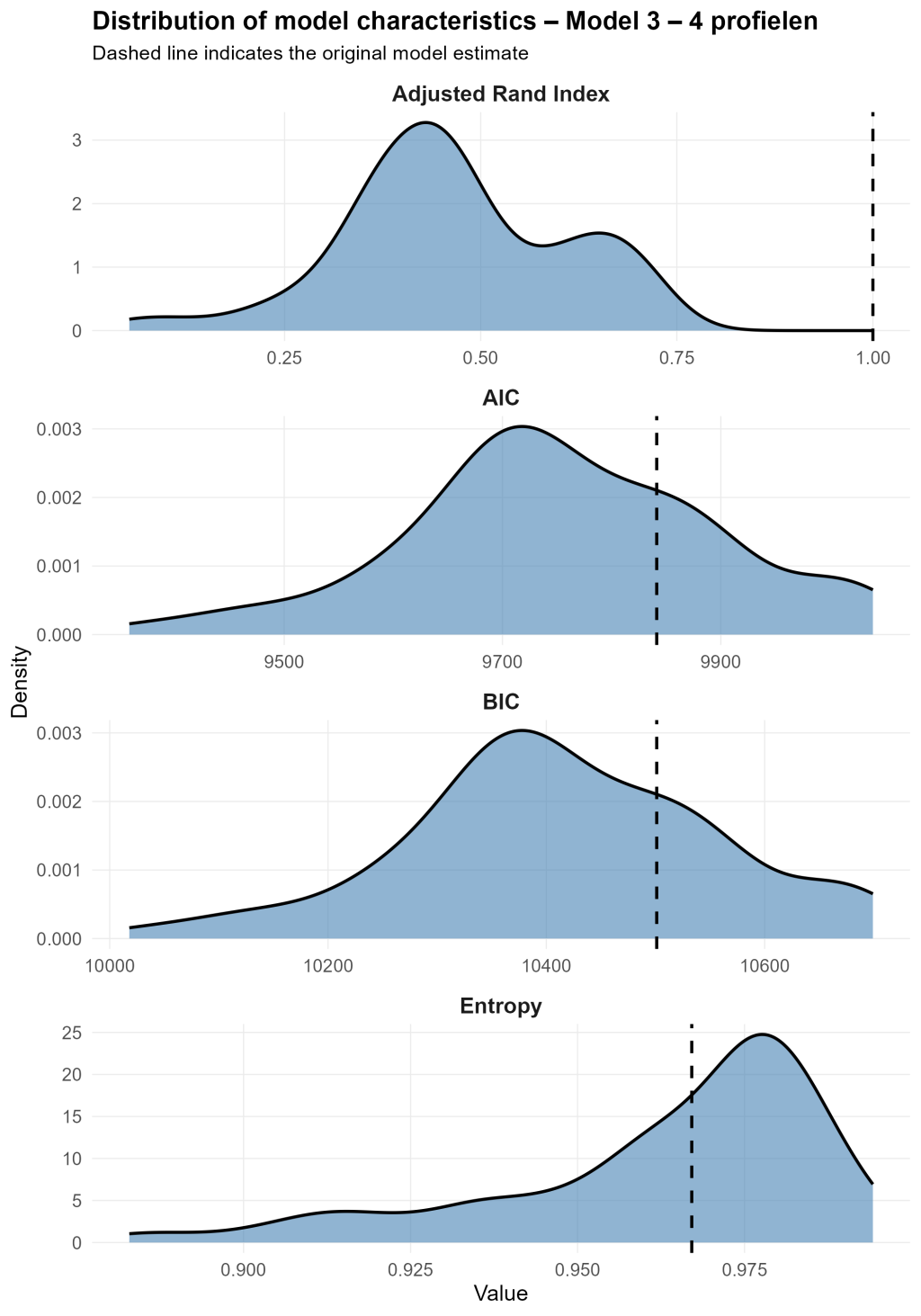
**

Bootstrap distributions of model characteristics for the selected latent profile model (Model 3 – 4 profiles). Density plots show the distribution of AIC, BIC, entropy, and the Adjusted Rand Index (ARI) across 100 bootstrap samples. The dashed vertical lines represent the corresponding estimates obtained from the model fitted to the original dataset. The bootstrap distributions provide an indication of the stability of the model fit and cluster classification across resampled datasets.
