# Supplementary material for: Identifying subgroups of individuals undergoing metabolic bariatric surgery based on behavioral and psychosocial factors: A latent profile analysis
Source: PLoS One. 2026 Jun 24;21(6):e0352252. doi: 10.1371/journal.pone.0352252 (PMC13293419; doi:10.1371/journal.pone.0352252)
Supplement: S1 Table — (DOCX) [file pone.0352252.s003.docx]

# **Supporting information**

**S1 Table.** **Fit indices for latent profile analyses**

| *Number of profiles* | *Log likelihood* | *AIC* | *BIC* | *SABIC* | *Entropy* | *n_min* | *n_max* | *BLRT_p* | *Convergence status* |
| --- | --- | --- | --- | --- | --- | --- | --- | --- | --- |
| **Model 1** | | | | | | | | | |
| 1 | \| -5781.8 \| \| --- \| \|  \| \|  \| \|  \| \|  \| \|  \| \|  \| \|  \| \|  \| \|  \| \|  \| \|  \| \|  \| \|  \| \|  \| | 11623.5 | 11731.7 | 11636.6 | 1 | \| 1 \| \| --- \| \|  \| \|  \| \|  \| \|  \| \|  \| \|  \| \|  \| \|  \| \|  \| \|  \| | \| 1 \| \| --- \| \|  \| \|  \| \|  \| \|  \| \|  \| | NA | True |
| 2 | -5357.0 | 10806.0 | 10971.9 | 10826.0 | 0.930 | 0.415 | 0.585 | NA | True |
| 3 | -5285.5 | 10695.0 | 10918.5 | 10722.0 | 0.848 | 0.235 | 0.397 | NA | True |
| 4 | -5154.6 | 10465.1 | 10746.4 | 10499.1 | 0.876 | 0.169 | 0.364 | NA | True |
| 5 | -5110.6 | 10409.2 | 10748.1 | 10450.1 | 0.861 | 0.162 | 0.235 | NA | True |
| 6 | -5085.9 | 10391.8 | 10788.5 | 10439.7 | 0.865 | 0.048 | 0.235 | NA | True |
| **Model 2** | | | | | | | | | |
| 1 | -5781.8 | 11623.5 | 11731.7 | 11636.6 | 1 | 1 | 1 | NA | True |
| 2 | -5183.2 | 10488.4 | 10708.4 | 10515.0 | 0.935 | 0.441 | 0.559 | NA | True |
| 3 | -5053.6 | 10291.1 | 10622.9 | 10331.2 | 0.892 | 0.276 | 0.412 | NA | True |
| 4 | NA | NA | NA | NA | NA | NA | NA | NA | False |
| 5 | NA | NA | NA | NA | NA | NA | NA | NA | False |
| 6 | NA | N | NA | NA | NA | NA | NA | NA | False |
| **Model 3** | | | | | | | | | |
| 1 | -4949.8 | 10169.7 | 10656.4 | 10228.4 | 1 | 1 | 1 | NA | True |
| 2 | -4878.4 | 10058.7 | 10603.2 | 10124.4 | 0.923 | 0.401 | \| 0.599 \| \| --- \| | \| 0.01 \| \| --- \| \|  \| \|  \| \|  \| \|  \| | True |
| 3 | -4831.3 | 9996.6 | 10598.7 | 10069.2 | 0.947 | \| 0.158 \| \| --- \| | \| 0.504 \| \| --- \| | 0.01 | True |
| **4** | **-4737.6** | **9841.3** | **10501.1** | **9920.9** | **0.967** | \| **0.132** \| \| --- \| | **0.449** | **0.01** | **True** |
| 5 | -4724.8 | 9847.7 | 10565.2 | 9934.2 | 0.950 | \| 0.055 \| \| --- \| | 0.412 | 0.42 | True |
| 6 | -4720.8 | 9871.6 | 10646.9 | 9965.1 | 0.869 | \| 0.044 \| \| --- \| | 0.279 | 1 | True |

AIC: Akaike Information Criterion; BIC: Bayesian Information Criterion; SABIC: Sample-size Adjusted BIC; BLRT: Bootstrapped Likelihood Ratio Test.
