# Supplementary material for: Identifying subgroups of individuals undergoing metabolic bariatric surgery based on behavioral and psychosocial factors: A latent profile analysis
Source: PLoS One. 2026 Jun 24;21(6):e0352252. doi: 10.1371/journal.pone.0352252 (PMC13293419; doi:10.1371/journal.pone.0352252)
Supplement: S2 Table — (DOCX) [file pone.0352252.s004.docx]

**S2 Table. Posterior probabilities**

| *Classes* | *Class* | *n* | *Mean_posterior* | *Min_posterior* |
| --- | --- | --- | --- | --- |
| **Model 1** | | | | |
| 1 | 1 | 272 | 1 | 1 |
| 2 | 1 | 159 | 0.982 | 0.510 |
| 2 | 2 | 113 | 0.982 | 0.543 |
| 3 | 1 | 108 | 0.926 | 0.512 |
| 3 | 2 | 100 | 0.977 | 0.585 |
| 3 | 3 | 64 | 0.867 | 0.470 |
| 4 | 1 | 99 | 0.924 | 0.466 |
| 4 | 2 | 60 | 0.951 | 0.522 |
| 4 | 3 | 67 | 0.893 | 0.505 |
| 4 | 4 | 46 | 0.962 | 0.436 |
| 5 | 1 | 46 | 0.842 | 0.505 |
| 5 | 2 | 58 | 0.956 | 0.448 |
| 5 | 3 | 60 | 0.907 | 0.528 |
| 5 | 4 | 64 | 0.883 | 0.378 |
| 5 | 5 | 44 | 0.975 | 0.804 |
| 6 | 1 | 42 | 0.862 | 0.505 |
| 6 | 2 | 57 | 0.963 | 0.507 |
| 6 | 3 | 13 | 0.905 | 0.560 |
| 6 | 4 | 64 | 0.868 | 0.505 |
| 6 | 5 | 52 | 0.872 | 0.456 |
| 6 | 6 | 44 | 0.972 | 0.747 |
| **Model 2** | | | | |
| 1 | 1 | 272 | 1 | 1 |
| 2 | 1 | 120 | 0.98 | 0.596 |
| 2 | 2 | 152 | 0.982 | 0.531 |
| 3 | 1 | 85 | 0.964 | 0.542 |
| 3 | 2 | 112 | 0.97 | 0.530 |
| 3 | 3 | 75 | 0.933 | 0.550 |
| **Model 3** | | | | |
| 1 | 1 | 272 | 1 | 1 |
| 2 | 1 | 163 | 0.978 | 0.511 |
| 2 | 2 | 109 | 0.975 | 0.505 |
| 3 | 1 | 137 | 0.984 | 0.632 |
| 3 | 2 | 92 | 0.973 | 0.564 |
| 3 | 3 | 43 | 0.965 | 0.506 |
| **4** | **1** | **122** | **0.987** | **0.646** |
| **4** | **2** | **53** | **0.952** | **0.546** |
| **4** | **3** | **36** | **0.994** | **0.965** |
| **4** | **4** | **61** | **0.999** | **0.939** |
| 5 | 1 | 15 | 0.878 | 0.525 |
| 5 | 2 | 56 | 0.964 | 0.571 |
| 5 | 3 | 112 | 0.971 | 0.507 |
| 5 | 4 | 29 | 0.977 | 0.802 |
| 5 | 5 | 60 | 0.997 | 0.904 |
| 6 | 1 | 76 | 0.845 | 0.516 |
| 6 | 2 | 57 | 0.954 | 0.464 |
| 6 | 3 | 12 | 0.931 | 0.607 |
| 6 | 4 | 40 | 0.787 | 0.487 |
| 6 | 5 | 27 | 0.939 | 0.591 |
| 6 | 6 | 60 | 0.991 | 0.538 |
