# Supplementary material for: Identifying subgroups of individuals undergoing metabolic bariatric surgery based on behavioral and psychosocial factors: A latent profile analysis
Source: PLoS One. 2026 Jun 24;21(6):e0352252. doi: 10.1371/journal.pone.0352252 (PMC13293419; doi:10.1371/journal.pone.0352252)
Supplement: S3 Table — (DOCX) [file pone.0352252.s005.docx]

**S3 Table. Bootstrap results for each run**

| **AIC** | **BIC** | **Entropy** | **ARI** |
| --- | --- | --- | --- |
| 9574,226315 | 10234,08809 | 0,962229553 | 0,661763257 |
| 9478,079054 | 10137,94083 | 0,975699219 | 0,718201402 |
| 9514,458293 | 10174,32007 | 0,976934716 | 0,657239331 |
| 10018,00655 | 10677,86833 | 0,97989566 | 0,372710719 |
| 9904,584538 | 10564,44632 | 0,959522033 | 0,399412259 |
| 9671,680462 | 10331,54224 | 0,979441892 | 0,126138699 |
| 9686,545311 | 10346,40709 | 0,968835262 | 0,418571989 |
| 9721,199086 | 10381,06086 | 0,967389071 | 0,555933706 |
| 9879,577975 | 10539,43975 | 0,974461987 | 0,449606929 |
| 9358,075276 | 10017,93705 | 0,981865171 | 0,662740076 |
| 9703,086347 | 10362,94813 | 0,984750248 | 0,671319757 |
| 9689,869467 | 10349,73125 | 0,972815244 | 0,448350181 |
| 9825,311259 | 10485,17304 | 0,971486949 | 0,51229805 |
| 9737,026415 | 10396,88819 | 0,961831156 | 0,609812872 |
| 9732,992708 | 10392,85449 | 0,925967398 | 0,374940089 |
| 9869,357847 | 10529,21962 | 0,994214306 | 0,436966601 |
| 9852,693751 | 10512,55553 | 0,954705105 | 0,483286979 |
| 9902,134364 | 10561,99614 | 0,914017057 | 0,300729617 |
| 9454,794751 | 10114,65653 | 0,965298757 | 0,452261501 |
| 9711,128346 | 10370,99012 | 0,982974463 | 0,361419896 |
| 9460,821785 | 10120,68356 | 0,982920885 | 0,712367181 |
| 9739,81694 | 10399,67872 | 0,972323791 | 0,616126243 |
| 9897,242887 | 10557,10467 | 0,888280261 | 0,052033125 |
| 9709,886806 | 10369,74858 | 0,978765831 | 0,675670074 |
| 9578,716006 | 10238,57778 | 0,947392395 | 0,384099216 |
| 9631,157402 | 10291,01918 | 0,98734862 | 0,539723131 |
| 9840,229876 | 10500,09165 | 0,911745165 | 0,381658887 |
| 9880,737656 | 10540,59943 | 0,958646591 | 0,448676111 |
| 10013,95368 | 10673,81546 | 0,989651747 | 0,292124143 |
| 9405,884779 | 10065,74656 | 0,977065776 | 0,702514703 |
| 9612,001385 | 10271,86316 | 0,979404432 | 0,633337336 |
| 9681,830667 | 10341,69245 | 0,980904402 | 0,448899547 |
| 10010,89073 | 10670,75251 | 0,986516214 | 0,474070891 |
| 9773,805815 | 10433,66759 | 0,917976267 | 0,3556658 |
| 9829,700813 | 10489,56259 | 0,937896683 | 0,345574072 |
| 9589,600657 | 10249,46244 | 0,962595025 | 0,397999393 |
| 9968,217789 | 10628,07957 | 0,976551461 | 0,416863298 |
| 9810,585162 | 10470,44694 | 0,98018689 | 0,475657832 |
| 9674,010235 | 10333,87201 | 0,960303938 | 0,428411272 |
| 9734,279473 | 10394,14125 | 0,972545349 | 0,446525852 |
| 10022,86253 | 10682,72431 | 0,987885371 | 0,43895546 |
| 9917,239747 | 10577,10153 | 0,992453338 | 0,493138717 |
| 9631,111373 | 10290,97315 | 0,978056839 | 0,386526464 |
| 9688,894806 | 10348,75658 | 0,987025278 | 0,398652275 |
| 9859,965681 | 10519,82746 | 0,978401661 | 0,478976665 |
| 9941,396004 | 10601,25778 | 0,90876031 | 0,245849654 |
| 9643,15409 | 10303,01587 | 0,935460169 | 0,684328218 |
| 9745,665171 | 10405,52695 | 0,986517685 | 0,399652353 |
| 9737,564797 | 10397,42657 | 0,976693832 | 0,476462324 |
| 9589,626724 | 10249,4885 | 0,965941478 | 0,439226877 |
| 10039,30929 | 10699,17107 | 0,978485575 | 0,477933241 |
| 9730,933587 | 10390,79537 | 0,97151699 | 0,634416179 |
| 9889,006573 | 10548,86835 | 0,975737133 | 0,425595683 |
| 9881,321506 | 10541,18328 | 0,96175601 | 0,458328744 |
| 9759,456399 | 10419,31818 | 0,963818075 | 0,510613463 |
| 10001,04665 | 10660,90843 | 0,933592079 | 0,584611408 |
| 9795,829302 | 10455,69108 | 0,882890886 | 0,363510094 |
| 9759,532548 | 10419,39433 | 0,985838021 | 0,543994218 |
| 9879,037349 | 10538,89913 | 0,963087803 | 0,530037245 |
| 9860,272473 | 10520,13425 | 0,973050572 | 0,408678219 |
| 9759,537902 | 10419,39968 | 0,905180526 | 0,237615688 |
| 9806,76912 | 10466,6309 | 0,899123555 | 0,297528949 |
| 9592,207511 | 10252,06929 | 0,975538644 | 0,645031103 |
| 9676,330608 | 10336,19239 | 0,933267988 | 0,452834316 |
| 9813,444523 | 10473,3063 | 0,967087984 | 0,486483743 |
| 10017,64821 | 10677,50999 | 0,946453297 | 0,437308269 |
| 9670,971035 | 10330,83281 | 0,97791527 | 0,375850045 |
| 9767,879488 | 10427,74127 | 0,935213182 | 0,079054625 |
| 9679,347043 | 10339,20882 | 0,970395805 | 0,458900306 |
| 9892,711612 | 10552,57339 | 0,969364641 | 0,491070515 |
| 9713,210319 | 10373,0721 | 0,96022381 | 0,596794673 |
| 9754,719128 | 10414,58091 | 0,960748523 | 0,543005989 |
| 9627,473369 | 10287,33515 | 0,924333836 | 0,286572284 |
| 9850,522866 | 10510,38464 | 0,95815359 | 0,188702418 |
| 10018,77047 | 10678,63225 | 0,979748986 | 0,457017951 |
| 9651,616091 | 10311,47787 | 0,983277111 | 0,702704455 |
| 9822,941566 | 10482,80334 | 0,973215972 | 0,447779366 |
| 9942,068121 | 10601,9299 | 0,978178563 | 0,401505059 |
| 9818,985676 | 10478,84745 | 0,950875313 | 0,384905133 |
| 9753,277065 | 10413,13884 | 0,976151421 | 0,352836801 |
| 9610,97702 | 10270,8388 | 0,956544224 | 0,661836434 |
| 9725,087983 | 10384,94976 | 0,958229724 | 0,451023533 |
| 9855,571821 | 10515,4336 | 0,913005672 | 0,387648215 |
| 9652,138056 | 10311,99983 | 0,975438484 | 0,712200062 |
| 9817,430772 | 10477,29255 | 0,94700246 | 0,352957457 |
| 9746,266431 | 10406,12821 | 0,92006966 | 0,430094041 |
| 9678,686817 | 10338,5486 | 0,949460073 | 0,226808716 |
| 9805,587508 | 10465,44929 | 0,978703376 | 0,594573971 |
| 9746,765918 | 10406,6277 | 0,94534396 | 0,369728484 |
| 9695,598406 | 10355,46018 | 0,977021652 | 0,698431396 |
| 9790,988908 | 10450,85069 | 0,98079182 | 0,495759598 |
| 9793,635991 | 10453,49777 | 0,94074591 | 0,332847442 |
| 9675,366451 | 10335,22823 | 0,935392259 | 0,341858073 |
| 9718,286949 | 10378,14873 | 0,991176575 | 0,336986143 |
| 9527,564802 | 10187,42658 | 0,992295414 | 0,351346917 |
| 9700,883287 | 10360,74506 | 0,984579334 | 0,596665572 |
| 9696,471679 | 10356,33346 | 0,954872237 | 0,447596255 |
| 9703,239236 | 10363,10101 | 0,982411907 | 0,636381223 |
| 9708,429772 | 10368,29155 | 0,981308481 | 0,616463254 |
| 9545,866073 | 10205,72785 | 0,974886094 | 0,695435884 |

AIC: Akaike Information Criterion; BIC: Bayesian Information Criterion; ARI: Adjusted Rand Index.
