# Supplementary material for: Identifying subgroups of individuals undergoing metabolic bariatric surgery based on behavioral and psychosocial factors: A latent profile analysis
Source: PLoS One. 2026 Jun 24;21(6):e0352252. doi: 10.1371/journal.pone.0352252 (PMC13293419; doi:10.1371/journal.pone.0352252)
Supplement: S4 Table — (DOCX) [file pone.0352252.s006.docx]

**S4 Table. Mean values per indicator variable by profile**

| *Indicator* | *Profile 1* | *Profile 2* | *Profile 3* | *Profile 4* |
| --- | --- | --- | --- | --- |
| DEBQ |  |  |  |  |
| Emotional eating | -0.08 | -0.07 | 0.15 | 0.13 |
| External eating | 0.04 | 0.01 | 0.05 | -0.12 |
| Restrained eating | -0.11 | 0.13 | 0.13 | 0.02 |
| EDE-Q | -0.27 | 0.00 | 0.18 | 0.43 |
| BDI | -0.46 | 0.17 | 0.15 | 0.68 |
| MSPSS | 0.24 | 0.14 | -0.12 | -0.52 |
| IWQOL-Lite | 0.42 | -0.25 | -0.05 | -0.60 |
| SF-36 |  |  |  |  |
| Physical functioning | 0.46 | -0.38 | 0.10 | -0.64 |
| Role limitations due to physical functioning | 0.83 | -1.00 | 0.36 | -1.01 |
| Role limitations due to emotional problems | 0.68 | 0.60 | -0.32 | -1.69 |
| Energy/fatigue | 0.50 | -0.23 | -0.20 | -0.67 |
| Emotional wellbeing | 0.30 | 0.17 | -0.34 | -0.55 |
| Social functioning | 0.52 | -0.18 | -0.18 | -0.78 |
| Bodily pain | 0.48 | -0.50 | 0.14 | -0.61 |
| General health | 0.42 | -0.27 | -0.11 | -0.54 |

DEBQ: Dutch eating behavior questionnaire; EDE-Q: Eating disorder examination questionnaire; BDI: Beck depression inventory; MSPSS: Multidimensional scale of perceived social support; IWQOL-Lite: Impact of weight on quality of life questionnaire; SF-36: Short form health survey.
