# Supplementary material for: Identifying subgroups of individuals undergoing metabolic bariatric surgery based on behavioral and psychosocial factors: A latent profile analysis
Source: PLoS One. 2026 Jun 24;21(6):e0352252. doi: 10.1371/journal.pone.0352252 (PMC13293419; doi:10.1371/journal.pone.0352252)
Supplement: S5 Table — (DOCX) [file pone.0352252.s007.docx]

**S5 Table. Effect size matrix**

| *Indicator* | *Profile 1 vs. 2* | *Profile 1 vs. 3* | *Profile 1 vs. 4* | *Profile 2 vs. 3* | *Profile 2 vs. 4* | *Profile 3 vs. 4* |
| --- | --- | --- | --- | --- | --- | --- |
| DEBQ |  |  |  |  |  |  |
| Emotional eating | -0.006706642 | -0.229697966 | -0.205220231 | -0.218582552 | -0.194390922 | 0.020144836 |
| External eating | 0.032982992 | -0.003486487 | 0.162678985 | -0.033650388 | 0.125021163 | 0.149388739 |
| Restrained eating | -0.246010718 | -0.22805839 | -0.121053767 | -0.003311128 | 0.109835066 | 0.098411661 |
| EDE-Q | -0.319385309 | -0.469631657 | -0.688460318 | -0.207765097 | -0.437639372 | -0.227387678 |
| BDI | -0.824760559 | -0.749273845 | -1.318580843 | 0.025754571 | -0.503598204 | -0.484747667 |
| MSPSS | 0.130058348 | 0.436760065 | 0.735560461 | 0.322739565 | 0.580580073 | 0.332872475 |
| IWQOL-Lite | 0.840440662 | 0.514311807 | 1.12522937 | -0.21715529 | 0.371742628 | 0.500804056 |
| SF-36 |  |  |  |  |  |  |
| Physical functioning | 1.052683332 | 0.435397536 | 1.253852546 | -0.519063045 | 0.256635015 | 0.707693119 |
| Role limitations due to physical functioning | 4.116307655 | 1.002761485 | 3.893681235 | -2.431996277 | 0.018461675 | 2.308566802 |
| Role limitations due to emotional problems | 0.516198897 | 13.01903369 | 8.451141809 | 4.11019235 | 5.76596512 | 3.419352307 |
| Energy/fatigue | 0.897827435 | 0.81920429 | 1.342569209 | -0.03653631 | 0.470309184 | 0.470105869 |
| Emotional wellbeing | 0.185047139 | 0.752646136 | 0.908056827 | 0.537593085 | 0.683234574 | 0.173165811 |
| Social functioning | 0.897656307 | 0.943922265 | 1.714206583 | -0.001613487 | 0.596811339 | 0.605729679 |
| Bodily pain | 1.189479703 | 0.399784068 | 1.272222012 | -0.685662708 | 0.124019081 | 0.765192663 |
| General health | 0.731124884 | 0.547965247 | 1.029345278 | -0.181429895 | 0.306287799 | 0.489068686 |

DEBQ: Dutch eating behavior questionnaire; EDE-Q: Eating disorder examination questionnaire; BDI: Beck depression inventory; MSPSS: Multidimensional scale of perceived social support; IWQOL-Lite: Impact of weight on quality of life questionnaire; SF-36: Short form health survey.
