# Supplementary material for: Identifying subgroups of individuals undergoing metabolic bariatric surgery based on behavioral and psychosocial factors: A latent profile analysis
Source: PLoS One. 2026 Jun 24;21(6):e0352252. doi: 10.1371/journal.pone.0352252 (PMC13293419; doi:10.1371/journal.pone.0352252)
Supplement: S6 Table — (DOCX) [file pone.0352252.s008.docx]

**S6 Table. Distribution of patients per profile by COVID-19 period**

|  | *Profile 1*  *(n = 122)* | *Profile 2*  *(n = 53)* | *Profile 3*  *(n = 36)* | *Profile 4*  *(n = 61)* |
| --- | --- | --- | --- | --- |
| Pre-COVID, n (%) | 7 (6) | 4 (8) | 1 (3) | 2 (3) |
| COVID, n (%) | 68 (56) | 30 (57) | 22 (61) | 41 (67) |
| Post-COVID, n (%) | 47 (39) | 19 (36) | 13 (36) | 18 (30) |
