# Supplementary material for: Identifying subgroups of individuals undergoing metabolic bariatric surgery based on behavioral and psychosocial factors: A latent profile analysis
Source: PLoS One. 2026 Jun 24;21(6):e0352252. doi: 10.1371/journal.pone.0352252 (PMC13293419; doi:10.1371/journal.pone.0352252)
Supplement: S7 Table — (DOCX) [file pone.0352252.s009.docx]

**S7 Table. Patient characteristics of the total population and stratified by COVID-19 period**

|  | *Total population*  *(n = 272)* | *Pre-COVID (1)*  *(n = 14)* | *COVID (2)*  *(n = 161)* | *Post-COVID (3)*  *(n = 97)* | *P* |
| --- | --- | --- | --- | --- | --- |
| Age, years, mean ± SD | 47 ± 10 | 46 ± 7 | 47 ± 10 | 48 ± 10 | 0.960 |
| Gender, n (%) |  |  |  |  | 0.830 |
| Female | 209 (76.8) | 10 (71.4) | 123 (76.4) | 76 (78.4) |  |
| Male | 63 (23.2) | 4 (28.6) | 38 (23.6) | 21 (21.6) |  |
| BMI, kg/m^2^, mean ± SD | 42.4 ± 4.8 | 41.4 ± 2.5 | 42.3 ± 4.5 | 42.6 ± 5.5 | 0.650 |
| Comorbidities, n (%) |  |  |  |  |  |
| T2DM | 41 (15.1) | 1 (7.1) | 22 (13.7) | 18 (18.6) | 0.642 |
| Hypertension | 102 (37.5) | 2 (14.3) | 64 (39.8) | 36 (37.1) | 0.168 |
| OSAS | 81 (29.8) | 3 (21.4) | 52 (32.3) | 26 (26.8) | 0.505 |
| Osteoarthrosis | 135 (49.6) | 6 (42.9) | 79 (49.1) | 50 (51.5) | 0.811 |
| GERD | 90 (33.1) | 3 (21.4) | 54 (33.5) | 33 (34.0) | 0.634 |
| Dyslipidemia | 222 (81.6) | 13 (92.9) | 130 (80.7) | 79 (81.4) | 0.532 |
| Highest level of completed education, n (%) |  |  |  |  | 0.863 |
| No degree | 7 (2.6) | 0 (0.0) | 5 (3.1) | 2 (2.1) |  |
| Primary school | 13 (4.8) | 2 (14.3) | 8 (5.0) | 3 (3.1) |  |
| High school - vocational education | 31 (11.4) | 1 (7.1) | 19 (11.8) | 11 (11.3) |  |
| High school - higher vocational education | 11 (4.0) | 0 (0.0) | 8 (4.9) | 3 (3.1) |  |
| Vocational education | 131 (48.2) | 8 (57.1) | 78 (48.4) | 45 (46.4) |  |
| Higher vocational education | 59 (21.7) | 3 (21.4) | 34 (21.1) | 22 (22.7) |  |
| University | 5 (1.8) | 0 (0.0) | 2 (1.2) | 3 (3.1) |  |
| Other | 15 (5.5) | 0 (0.0) | 7 (4.3) | 8 (8.2) |  |
| Employment, n (%) |  |  |  |  | 0.546 |
| Student | 3 (1.1) | 0 (0.0) | 3 (1.9) | 0 (0.0) |  |
| Working | 183 (67.3) | 10 (71.4) | 107 (66.5) | 66 (68.0) |  |
| Unemployed | 78 (28.7) | 4 (28.6) | 48 (29.8) | 26 (26.8) |  |
| Other | 8 (2.9) | 0 (0.0) | 3 (1.9) | 5 (5.2) |  |
| Marital status, n (%) |  |  |  |  | 0.776 |
| Single | 36 (13.2) | 1 (7.1) | 23 (14.3) | 12 (12.4) |  |
| In a relationship (not cohabiting) | 8 (2.9) | 0 (0.0) | 6 (3.7) | 2 (2.1) |  |
| Cohabiting | 35 (12.9) | 3 (21.4) | 19 (11.8) | 13 (13.4) |  |
| Married | 175 (64.3) | 10 (71.4) | 101 (62.7) | 64 (66.0) |  |
| Divorced | 14 (5.1) | 0 (0.0) | 8 (5.0) | 6 (6.2) |  |
| Widowed | 4 (1.5) | 0 (0.0) | 4 (2.5) | 0 (0.0) |  |
| Smoking, n (%) | 23 (8.5) | 2 (14.3) | 11 (6.8) | 10 (10.3) |  |
| Alcohol consumption in units/week, n (%) |  |  |  |  | 0.138 |
| 0 | 136 (50) | 6 (42.9) | 76 (47.2) | 54 (55.7) |  |
| 1 – 7 | 126 (46.3) | 7 (50.0) | 79 (49.1) | 40 (41.2) |  |
| 8 – 14 | 7 (2.6) | 0 (0.0) | 5 (3.1) | 2 (2.1) |  |
| 15 – 21 | 2 (0.7) | 1 (7.1) | 0 (0.0) | 1 (1.0) |  |
| > 21 | 1 (0.4) | 0 (0.0) | 1 (0.6) | 0 (0.0) |  |

SD: Standard deviation; NA: Not applicable; BMI: Body Mass Index; T2DM: Type 2 diabetes mellitus; OSAS: Obstructive sleep apnea syndrome; GERD: Gastro-esophageal reflux disease.
