# Supplementary material for: Identifying subgroups of individuals undergoing metabolic bariatric surgery based on behavioral and psychosocial factors: A latent profile analysis
Source: PLoS One. 2026 Jun 24;21(6):e0352252. doi: 10.1371/journal.pone.0352252 (PMC13293419; doi:10.1371/journal.pone.0352252)
Supplement: S8 Table — (DOCX) [file pone.0352252.s010.docx]

**S8 Table. Behavioral and psychosocial-based metrics of the total population and stratified by COVID-19 period**

|  | *Total population*  *(n = 272)* | *Pre-COVID (1)*  *(n = 14)* | *COVID (2)*  *(n = 161)* | *Post-COVID (3)*  *(n = 97)* | *P* | *Post-hoc test*  *Tukey’s HSD* |
| --- | --- | --- | --- | --- | --- | --- |
| DEBQ, mean ± SD |  |  |  |  |  |  |
| Emotional eating | 2.1 ± 0.7 | 1.9 ± 0.7 | 2.1 ± 0.7 | 2.2 ± 0.8 | 0.318 | NA |
| External eating | 2.6 ± 0.6 | 2.7 ± 0.7 | 2.5 ± 0.6 | 2.6 ± 0.7 | 0.724 | NA |
| Restrained eating | 3.0 ± 0.6 | 3.0 ± 0.6 | 3.0 ± 0.6 | 2.9 ± 0.7 | 0.098 | NA |
| EDE-Q, mean ± SD | 2.1 ± 0.9 | 2.0 ± 1.2 | 2.2 ± 0.9 | 1.9 ± 0.9 | 0.198 | NA |
| BDI, mean ± SD | 8.6 ± 5.7 | 7.7 ± 5.1 | 9.2 ± 5.9 | 7.8 ± 5.3 | 0.133 | NA |
| MSPSS, mean ± SD | 71.0 ± 13.8 | 67.1 ± 15.3 | 71.6 ± 13.3 | 70.5 ± 14.4 | 0.482 | NA |
| IWQOL-Lite, mean ± SD | 62.3 ± 18.0 | 64.4 ± 17.1 | 60.0 ± 17.8 | 65.7 ± 18.1 | 0.043 | 3 > 2 |
| SF-36, mean ± SD |  |  |  |  |  |  |
| Physical functioning | 58.8 ± 21.7 | 58.6 ± 19.3 | 58.1 ± 22.1 | 60.1 ± 21.6 | 0.769 | NA |
| Role limitations due to physical functioning | 55.7 ± 40.2 | 50.0 ± 43.9 | 55.6 ± 41.4 | 56.7 ± 38.1 | 0.844 | NA |
| Role limitations due to emotional problems | 76.8 ± 34.2 | 88.1 ± 24.8 | 74.1 ± 35.7 | 79.7 ± 32.5 | 0.200 | NA |
| Energy/fatigue | 53.1 ± 18.8 | 60.0 ± 18.8 | 51.6 ± 18.6 | 54.5 ± 19.0 | 0.180 | NA |
| Emotional wellbeing | 69.6 ± 9.0 | 68.9 ± 6.5 | 68.7 ± 9.7 | 71.2 ± 8.0 | 0.100 | NA |
| Social functioning | 77.0 ± 23.4 | 88.4 ± 13.4 | 75.9 ± 24.0 | 77.2 ± 23.2 | 0.160 | NA |
| Bodily pain | 58.1 ± 24.3 | 56.1 ± 19.9 | 57.5 ± 24.8 | 59.5 ± 24.2 | 0.772 | NA |
| General health | 48.1 ± 18.8 | 50.0 ± 18.2 | 48.4 ± 19.6 | 47.4 ± 17.6 | 0.847 | NA |

DEBQ: Dutch eating behavior questionnaire; SD: Standard deviation; EDE-Q: Eating disorder examination questionnaire; BDI: Beck depression inventory; MSPSS: Multidimensional scale of perceived social support; IWQOL-Lite: Impact of weight on quality of life questionnaire; SF-36: Short form health survey.
