# Supplementary material for: Identifying subgroups of individuals undergoing metabolic bariatric surgery based on behavioral and psychosocial factors: A latent profile analysis
Source: PLoS One. 2026 Jun 24;21(6):e0352252. doi: 10.1371/journal.pone.0352252 (PMC13293419; doi:10.1371/journal.pone.0352252)
Supplement: S9 Table — (DOCX) [file pone.0352252.s011.docx]

**S9 Table. Baseline characteristics of the study population and the LABS cohort**

|  | *Total population*  *(n = 272)* | *LABS cohort*  *RYGB*  *(n = 1738)* | *LABS cohort*  *AGB*  *(n = 610)* |
| --- | --- | --- | --- |
| Age, years, median (IQR) | 49 (41-54) | 45 (37-54) | 48 (37-56) |
| Gender, n (%) |  |  |  |
| Female | 209 (77) | 1389 (80) | 465 (76) |
| Male | 63 (23) | 349 (20) | 145 (24) |
| BMI, kg/m^2^, median (IQR) | 41 (39-45) | 47 (42-52) | 44 (40-48) |
| Comorbidities, n (%) |  |  |  |
| T2DM | 41 (15) | 583/1646* (35) | 164/569* (29) |
| Hypertension | 102 (38) | 1159/1682* (69) | 367/585* (63) |
| Smoking, n (%) | 23 (9) | 250/1734* (14) | 56/610* (9) |

RYGB: Roux-en-Y Gastric Bypass; AGB: Adjustable gastric band.

* No./Total (%)
